# Molecular Mechanism of Astragaloside IV in Improving Endothelial Dysfunction of Cardiovascular Diseases Mediated by Oxidative Stress

**DOI:** 10.1155/2021/1481236

**Published:** 2021-11-19

**Authors:** Peipei Meng, Rui Yang, Fenjun Jiang, Jianbo Guo, Xinyu Lu, Tao Yang, Qingyong He

**Affiliations:** ^1^Guang'anmen Hospital, China Academy of Chinese Medical Sciences, Beijing, China; ^2^Key Laboratory of Chinese Internal Medicine of Ministry of Education and Beijing, Dongzhimen Hospital, Beijing, China; ^3^Department of Neurosurgery, Sanbo Brain Hospital, Capital Medical University, Beijing, China; ^4^Faculty of Medicine, The University of Hong Kong, Hong Kong, China; ^5^Beijing University of Chinese Medicine, No. 11, North Tird Ring East Road, Beijing, China

## Abstract

Endothelial dysfunction, induced by oxidative stress, is an essential factor affecting cardiovascular disease. Uncoupling of endothelial nitric oxide synthase (eNOS) leads to a decrease in nitric oxide (NO) production, an increase in reactive oxygen species (ROS) production, NO consumption, and NO synthesis. As a main active ingredient of astragalus, astragaloside IV can reduce the apoptosis of endothelial cells during oxidative stress. This review is aimed at exploring the mechanism of astragaloside IV in improving oxidative stress-mediated endothelial dysfunction relevant to cardiovascular diseases. The findings showed that the astragaloside IV can prevent or reverse the uncoupling of eNOS, increase eNOS and NO, and enhance several activating enzymes to activate the antioxidant system. In-depth validation and quantitative experiments still need to be implemented.

## 1. Introduction

Cardiovascular disease, which is the leading cause of morbidity and mortality in the world [1, 2], largely affects human health and economy. Endothelial dysfunction is defined as a state of impaired vasodilation and proinflammatory and prothrombosis, mainly caused by the oxidative stress related to the increase of ROS production and the reduction of the bioavailability of NO in blood vessels and myocardium [3, 4]. Preventing endothelial dysfunction by improving oxidative stress and increasing endothelial NO production is regarded as a treatment strategy for cardiovascular diseases.

Astragaloside IV is one of the main chemical components of the traditional Chinese medicine astragalus. In the latest edition of the Chinese Pharmacopoeia [5], astragalus belongs to the dried rhizome of the legume Astragalus mongolia or Astragalus capsularis. As the main active component polysaccharide of Astragalus membranaceus, astragaloside IV is a lanolin alcohol-shaped tetracyclic triterpene saponins extracted from Astragalus membranaceus. It has poor water solubility and is soluble in methanol, ethanol, and acetone. Its molecular formula is C41H68O14, and its molecular weight is 784.97 Da [6]. According to modern pharmacological research, astragaloside IV has the effects of protecting cardiovascular and cerebrovascular, anti-inflammatory, immune regulation, antioxidation, and antiapoptosis [7–9]. However, the exactly mechanism of astragaloside IV in improving endothelial dysfunction mediated by oxidative stress remains unknown, especially of antioxidant factors, intracellular processes, and cardiovascular protection.

## 2. Endothelial Dysfunction Mediated by Oxidative Stress

Numerous factors participate in the development of endothelial dysfunction, and oxidative stress is one of the most critical factors. Oxidative stress is mediated by reactive oxygen species. When the production and elimination of oxidative substances in the body or cells are out of balance or excessive intake of exogenous oxidizing substances, it will cause ROS to accumulate in cells and trigger an oxidative reaction state. ROS includes unstable free radicals, oxygen ions, and peroxides and is a by-product of aerobic respiration and various metabolic processes in cells. In most cell types, mitochondria are the main driving force for the production of intracellular oxidants. In addition, other endogenous oxidases (such as nicotinamide adenine dinucleotide phosphate oxidase, xanthine oxidase, and epoxy synthase) and other organelles (peroxisomes and endoplasmic reticulum) all contribute to the production of intracellular ROS [10].

Oxidative stress causes endothelial dysfunction through a variety of mechanisms. (1) The uncoupling of eNOS leads to a decrease in NO production and an increase in ROS. In this process, the oxidation of NADPH oxidase and BH4 played a key role [11]. As an essential condition for NO synthesis, the cofactor BH4 is extremely easy to be oxidized. When eNOS lacks the cofactor BH4 required by it, it will uncouple and reduce NO and generate ROS [12, 13]. NADPH oxidase is a multiunit enzyme complex. At physiological concentrations, NADPH oxidase-derived ROS acts as a signal sensor to mediate endothelial cell angiogenesis and migration through vascular endothelial growth factor and small G protein Rac1 [14]. Under pathological conditions, it is an important source of ROS in endothelial cells. The process of ROS release mediated by NADPH oxidase is also called oxidative burst [15]. (2) Increase in NO consumption: superoxide reacts strongly with vascular NO to form toxic peroxynitrite, which leads to changes in cell membrane structure, damages endothelial cell function, and further reduces the availability of NO. (3) The reduction of NO synthesis raw materials leads to the reduction of NO synthesis: arginase can compete with eNOS for the substrate, and the increase of arginase activity leads to the decrease of eNOS substrate L-arginine, which reduces the synthesis and secretion of NO [16]. (4) Expression stimulation of endothelial vasoconstrictors: ET-1, PG and angiotensin-II, and other vasoconstrictor factors are upregulated under oxidative stress conditions, which can strongly inhibit NO-mediated vasodilation and promote vasoconstriction, promote blood coagulation, and affect endothelial function [17].

## 3. Effects of Astragaloside IV on Oxidative Stress

### 3.1. Antioxidant Factors

Superoxide dismutase (SOD), glutathione peroxidase (GSH-Px), and catalase (CAT) are the key antioxidant enzymes against oxidative stress. Antioxidant enzymes reflect oxidative stress. Horizontally, they work by removing ROS. Studies have shown that astragaloside IV can increase the expression levels of SOD, GSH-Px, and CAT in the hippocampus of rats with oxidative stress, neuroinflammation, and cognitive impairment induced by A*β*1-42 [18]. It can also protect H_2_O_2_-induced oxidative damage by activating the NFE2L2-ARE signaling pathway [19]. NFE2L2 is a transcription factor susceptible to oxidative stress. It binds to the ARE on the chromatin and promotes the transcription of various antioxidant enzymes [20]. Under normal circumstances, NFE2L2 binds to Kelch-like ECH-related protein 1 (Keap1) in the cytoplasm, making it unable to transfer to the nucleus for transcription. Under oxidative conditions, NFE2L2 dissociates from Keap1 and transfers to the nucleus and combines with ARE to promote SOD, CAT, GSH-Px, and other antioxidant enzyme (such as heme oxygenase-1 (HMOX1) and NADPH quinone dehydrogenase 1 (NQO1)) expression [21]. Astragaloside IV can promote NFE2L2 to enter the nucleus, thereby promoting the transcription of antioxidant enzymes, thereby enhancing the ability of small intestinal epithelial cells to remove ROS and protecting them from oxidative damage. In addition, astragaloside IV can reduce the oxidative damage of renal tubular epithelial cells caused by PM2.5 by regulating the Keap1-Nrf2-ARE signaling pathway [22]. Human umbilical vein endothelial cell culture experiments also showed that astragaloside IV significantly improved the inactivation of the NO-eNOS signaling pathway induced by homocysteine by reducing ROS and increasing the activity of SOD [23].

### 3.2. Intracellular Sources

#### 3.2.1. Reduce the ROS Level

Under normal circumstances, the production and elimination of ROS in the body are balanced. In hypoxia, the stressful production of ROS exceeds the scavenging ability of the endogenous antioxidant system, resulting in an imbalance of ROS metabolism. Excessive accumulation of ROS is the chief culprit in inducing a series of oxidative damage reactions [24]. Therefore, whether it can effectively reduce ROS in the body is a key point for improving endothelial dysfunction caused by oxidative stress. One study [25] found that astragaloside IV can significantly reduce doxorubicin- (DOX-) induced ROS production and the release of LDH, CK-MB, and cytochrome C and restore mitochondrial function. The other study [26] also proved that astragaloside IV could inhibit the production of ROS and NADPH by upregulating PGC-1*α* and TFAM and promote mitochondrial autophagy and mitochondrial biogenesis and use its antioxidant activity to help protect damaged mitochondria.

#### 3.2.2. Prevent or Reverse eNOS Uncoupling

Among many enzymatic systems capable of producing O_2_^·-^, NADPH oxidase and uncoupled eNOS are the main sources of O_2_^·-^ in endothelial cells. O_2_^·-^ generated by NADPH oxidase may trigger eNOS uncoupling [27]. Two studies [28, 29] showed that astragaloside IV can improve the oxidative stress induced by H_2_O_2_ and isoproterenol (Iso), and its effect may be through the inhibition of NADPH oxidase-ROS-NF. Uncoupling of *κ*B pathway and weakening eNOS is achieved. The results of the study showed that astragaloside IV inhibited the production of O_2_^·-^ in the rat aorta and increased the ratio of eNOS dimer/monomer, the key cofactor BH4 content, and the production of NO in the aorta.

It is known that EPC exposure to BaP (the main component of tobacco smoke) can induce oxidative stress by inducing ROS generation and activating NF-*κ*B, leading to endothelial dysfunction [30]. One study explored the protective effect of astragaloside IV on endothelial progenitor cell dysfunction induced by BaP (the main component of tobacco smoke). They found that astragaloside IV reduces ROS production through the RAGE pathway to reduce oxidative stress. RAGE is a receptor for advanced glycation end products (AGEs). Under normal circumstances, the level of RAGE expressed on EPCs is low. Under pathological conditions, the proinflammatory factor TNF-*α* activates the NF-*κ*B site in the RAGE promoter to induce endothelial RAGE expression [31]. The increased combination of AGEs and RAGE can promote ROS generation and cause oxidative stress [32]. Besides, astragaloside IV was proven to downregulate the expression of ROS and TNF-*α*, increase SOD activity, and prevent EPC dysfunction caused by BaP-mediated oxidative stress [33].

#### 3.2.3. Increase the eNOS and NO Levels

Astragaloside IV can effectively improve endothelial-dependent relaxation impairment and can increase endothelial nitric oxide synthase (eNOS) and nitric oxide (NO) levels both in vivo and in vitro [34]. One study [35] showed that astragaloside IV significantly improved aortic endothelial function, the phosphorylation of Akt at Ser473, and the dephosphorylation of eNOS at Thr495 could regulate the PI3K/Akt/eNOS signaling pathway and increase eNOS expression and NO production. Moreover, a high-fructose/high-fat diet will increase oxidative stress and reduce the ratio of myocardial eNOS dimers, thereby weakening the production of NO and reducing the production of cyclic guanosine phosphate (cGMP). Astragaloside IV can effectively promote the production of NO and cGMP in the myocardium and improve diastolic function. The underlying mechanism is related to reducing oxidative stress and activating the endothelial eNOS/NO/cGMP pathway [36]. It is notable that the JAK2/STAT3 pathway is involved in oxidative stress [37]. Astragaloside IV pretreatment can inhibit 6-OHDA's oxidative stress damage to PC12 cells, and its molecular mechanism is related to the activation of the JAK2/STAT3 signaling pathway [38]. Based on activating JAK2/STAT3 and ERK1/2 signaling pathways, astragaloside IV can upregulate endothelial eNOS expression and NO production [39].

## 4. Protective Effects of Astragaloside IV on Cardiovascular Disease

### 4.1. Endothelial Cell Dysfunction and Cardiovascular Disease

Endothelial cell dysfunction is the first step to cause cardiovascular disease and is closely related to many diseases such as hypertension and coronary heart disease [40]. Vascular endothelial cells are a single layer of long and flat cells on the long axis of the vascular lumen. They are an essential endocrine organ of the human body. They can produce and release a variety of endothelial-derived vasoactive substances, including nitric oxide, prostacyclin, and endothelial-derived relaxation factors represented by endothelial hyperpolarizing factors and endothelium-derived contractile factors, mainly endothelin-1 and prostaglandins. These vasoactive molecules maintain the balance of vasomotion and contraction by regulating the tension and diameter of blood vessels and participate in the activation or inactivation of a variety of cardiovascular enzymes [41]. It plays an essential role in maintaining blood vessel homeostasis and repair, regulating molecular transmission between blood and tissues, ensuring oxygen supply and metabolic requirements of tissues and organs, and participating in vascular structure remodeling [42–44]. Under unbalanced conditions, there will be a reduction in the production and/or availability of NO and an imbalance in the ratio of endothelial-derived vasodilators and vasoconstrictors, which ultimately leads to endothelial dysfunction. Among them, nitric oxide is a key factor that regulates endothelial function. It is not only a very important endothelial vasodilator but also inhibits the adhesion of white blood cells to the blood vessel wall, inhibits platelet aggregation and adhesion, and inhibits the formation of vascular lesions—the key process [45].

### 4.2. Protective Mechanism of Astragaloside IV

Cardiovascular protection is embodied in protecting myocardial cells, reducing myocardial ischemia-reperfusion injury, and regulating energy metabolism. Protect cardiomyocytes through antioxidant properties [46]. Astragaloside IV can inhibit the TGF*β*1 signaling pathway by affecting the content of angiotensin-II and aldosterone to improve cardiac structure remodeling, myocardial hypertrophy, and myocardial fibrosis, thereby enhancing cardiac function [47]. The improvement of metabolic regulation is to influence metabolic regulation by inhibiting liver glycogen phosphorylase and glucose-6-phosphatase, reducing blood sugar, regulating fat metabolism, and regulating energy metabolism [48].

The lack of NO can also accelerate vascular endothelium damage by blocking the proliferation of vascular endothelial growth factors [49]. Endothelial cells regulate the synthesis and secretion of NO through a cytochrome p450 reductase-like enzyme in the cell-nitric oxide synthase. Tetrahydrobiopterin acts as a cofactor in this process, and L-arginine oxidation acts as a substrate [50]. When endothelial dysfunction, the secreted NO decreases, which can lead to the performance of endothelial dysfunction such as impaired vasodilation, platelet activation, and thrombosis, increased endothelial permeability, and adhesion of white blood cells to the blood vessel wall, which ultimately leads to vascular damage.

## 5. Discussion

**Figure 1:**
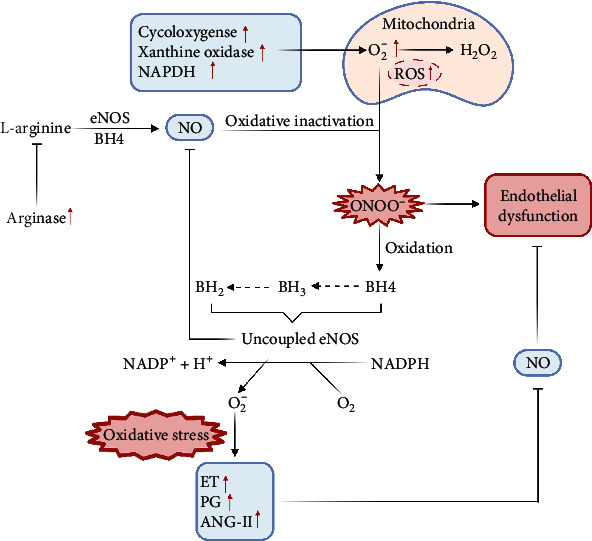
Endothelial dysfunction mediated by oxidative stress.

This study discusses the molecular mechanism of astragaloside IV, an active ingredient of the traditional Chinese medicine Astragalus membranaceus, on endothelial dysfunction mediated by oxidative stress. Oxidative stress occupies a leading role in the process of endothelial dysfunction, and the reduction of NO secretion plays a vital role in vascular endothelial damage. The accumulation of reactive oxygen species in cells triggers an oxidation reaction, and mitochondria are the main driving force of oxidants in cells. Oxidative stress causes endothelial dysfunction by various mechanisms, and increased endothelial NO secretion can effectively prevent endothelial dysfunction. The oxidation of NADPH oxidase and BH4 plays a key role in the uncoupling of eNOS, which leads to the decrease of NO production and the increase of ROS; the increase of NO consumption, the reduction of synthetic raw materials, and the expression of vasoconstrictor factors can all lead to endothelial dysfunction (Figure 1).

**Figure 2:**
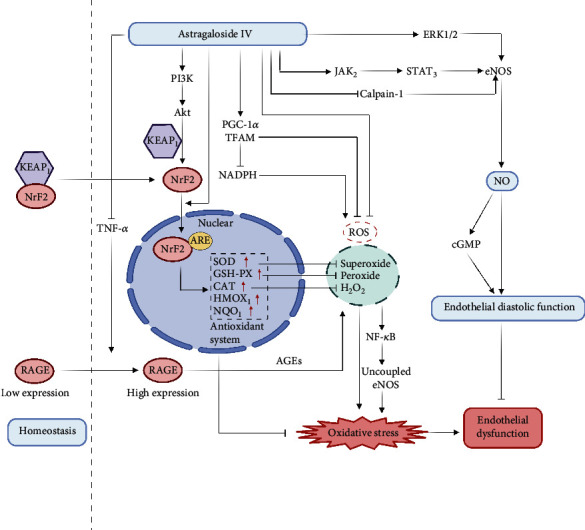
The general mechanisms involved in astragaloside IV's improvement on endothelial dysfunction mediated by oxidative stress.

Astragaloside IV improves endothelial dysfunction mediated by oxidative stress through a variety of mechanisms. Astragaloside IV reduces ROS production induced by DOX, upregulates PGC-1*α* and TFAM to inhibit the production of NADPH, and reduces the excessive accumulation of reactive oxygen species in the body to help protect damaged mitochondria and restore the function of mitochondria to balance ROS. Astragaloside IV inhibits the NADPH oxidase-ROS-NF-*κ*B pathway and weakens the uncoupling of eNOS to slow down the O_2_^·-^ enzymatic reaction, reduces the generation of ROS through the RAGE pathway, and downregulates the expression of ROS and TNF-*α* to prevent BaP mediation oxidative stress response. Antioxidant enzymes reflect the level of oxidative stress. Astragaloside IV protects H_2_O_2_-induced oxidative damage by activating the NFE2L2-ARE signaling pathway, and it also reduces intracellular oxidative damage by regulating the Keap1-Nrf2-ARE signaling pathway. Astragaloside IV activates the JAK2/STAT3 and ERK1/2 signaling pathways, upregulates the expression of endothelial eNOS and the production of NO, and improves endothelial dysfunction (Figure 2).

Calpain-1 exists in endothelial cells and participates in the formation of endothelial dysfunction [51]. Nie et al. [52] found that astragaloside IV can improve the endothelial dysfunction of the thoracic aorta in diabetic rats by reducing oxidative stress and downregulating calpain-1. In addition, astragaloside IV can significantly promote HUVEC cell proliferation and reduce apoptosis to improve endothelial dysfunction caused by a hypoxic environment [53]. The study of Wang et al. [54] showed that astragaloside IV could also affect the balance between the ubiquitination of hypoxia-inducible factor-1*α* (HIF-1*α*) and the modification of small ubiquitin-related modifiers (SUMO) in the nucleus. Inducing vascular endothelial cells to produce SUMO1 continuously stabilizes the HIF-1*α*/VEGF pathway, improves endothelial dysfunction under hypoxic conditions, and promotes angiogenesis.

The protection of the brain is reflected in protecting cerebral vascular and brain nerve, xanthine via anti-inflammatory, and antioxidation, inhibiting matrix metalloproteinase, and activating JNK and other paths to protect the cerebrovascular blood vessels. For Parkinson's research [55], Astragalus Gampta is delayed by inhibiting mitochondrial dysfunction and ROS and has a delaying effect for MPP+-induced SH-SY5Y cell death. Astragaloside IV by weakening the neuronal apoptosis in hippocampus inhibition of brain neurological lesions protects the cerebral nerve [56]. Astragaloside IV can improve metabolic regulation by inhibiting hepatic candied phosphoryase and glucose-6-phosphatase, reduce blood sugar, regulate fat metabolism, and regulate energy metabolism [57]. Astragaloside IV can reduce blood glucose, insulin, and blood lipid levels by lowering inflammatory genes of gestational diabetic mice, reducing CAMP accumulation in the liver to reduce hepatic glycogen ingeny [58]. Astragaloside IV has an increase in the expression of *α*7nachr in hypothalamus and adipose tissue, which has inhibitory effect on the central and peripheral inflammatory response, and improves leptin resistance, which has a good effect on obese hypertension [59].

The endothelial dysfunction caused by oxidative stress is mainly caused by the increase in ROS production and the decrease in the bioavailability of NO in blood vessels and myocardium. A variety of cardiovascular disease risk factors, such as hypertension, diabetes, and atherosclerosis, can lead to a sharp increase in reactive ROS in the blood vessel wall, leading to the oxidation of DNA, protein, carbohydrates, lipids, and other biological macromolecules and ultimately causes oxidative stress. Therefore, the prevention of endothelial dysfunction by reducing oxidative stress and increasing endothelial NO production is regarded as a reasonable treatment strategy for cardiovascular diseases.

## 6. Conclusion and Outlook

Astragaloside IV improves oxidative stress-mediated endothelial dysfunction mainly by reducing the ROS that induce oxidative stress, preventing or reversing the uncoupling of eNOS, and increasing eNOS and NO and a variety of activating enzymes to activate the antioxidant system and other molecular mechanisms, which are clinical vascular endothelial dysfunction diseases provide a certain basis. The research on the drug development of astragaloside IV and the mechanism of action of other system diseases is still moving forward, and further exploration is needed.
